# Leveraging e-health for enhanced cancer care service models in middle-income contexts: Qualitative insights from oncology care providers

**DOI:** 10.1177/20552076241237668

**Published:** 2024-03-13

**Authors:** Samar J Melhem, Shereen Nabhani-Gebara, Reem Kayyali

**Affiliations:** 1 Department of Pharmacy, School of Life Sciences, Pharmacy and Chemistry, 4264 Kingston University London, Kingston upon Thames, Surrey, UK; 2 Department of Biopharmaceutics and Clinical Pharmacy, School of Pharmacy, 54658 The University of Jordan, Amman, Jordan

**Keywords:** e-health, digital, telemedicine, LMICs, opportunities, challenges, e-reforms, service delivery, improvement, cancer care, COVID-19, qualitative

## Abstract

**Background:**

Global cancer research has predominantly favoured high-income countries (HICs). The unique challenges in low- and middle-income countries (LMICs) demand tailored research approaches, accentuated further by the disparities highlighted during the COVID-19 pandemic.

**Aim and objectives:**

This research endeavoured to dissect the intricacies of cancer care in LMICs, with Jordan serving as a case study. Specifically, the study aimed to conduct an in-depth analysis of the prevailing cancer care model and assess the transformative potential of eHealth technologies in bolstering cancer care delivery.

**Methods:**

Utilising a qualitative methodology, in-depth semi-structured interviews with oncology healthcare professionals were executed. Data underwent inductive thematic analysis as per Braun and Clarke's guidelines.

**Results:**

From the analysed data, two dominant themes surfaced. Firstly, “The current state of cancer care delivery” was subdivided into three distinct subthemes. Secondly, “Opportunities for enhanced care delivery via e-health” underscored the urgency of digital health reforms.

**Conclusion:**

The need to restrategise cancer care in LMICs is highlighted by this study, using the Jordanian healthcare context as a reference. The transformative potential of e-health initiatives has been illustrated. However, the relevance of this study might be limited by its region-specific approach. Future research is deemed essential for deeper exploration into the integration of digital health within traditional oncology settings across diverse LMICs, emphasising the significance of telemedicine in digital-assisted care delivery reforms.

## Introduction

Global cancer research has predominantly catered to the perspectives and concerns of high-income countries (HICs).^1^ HICs have distinct oncological datasets, research paths and health infrastructures in comparison to low- and middle-income countries (LMICs).^1,2^ Relying heavily on outcomes from HICs might not meet the unique needs of LMICs, emphasising the need for research aligned with LMIC contexts.^3^ Furthermore, transposing high-cost interventions, common in HICs, often faces challenges in LMICs due to differences in resources, infrastructure and socio-cultural contexts.^4^ In HICs, robust internet connectivity, stable electricity supply and widespread access to advanced technologies enable seamless integration of digital health solutions.^5^ In contrast, LMICs often encounter significant challenges related to infrastructure, such as inconsistent and limited internet connectivity, frequent power outages and restricted access to contemporary digital technologies, especially in rural and underserved areas.^5,6^ The significant digital divide between HICs and LMICs impedes the effective implementation and scalability of digital health initiatives in LMICs, exacerbating already existing healthcare disparities. Addressing these disparities necessitates strategic investments and collaborative efforts to improve LMICs’ digital infrastructure, ensuring equitable access to digital health advances.^7^

The disproportionate burden of cancer LMICs is a major global health concern. LMICs account for approximately 70% of global cancer deaths, despite having lower incidence rates than HICs.^8^ This disparity highlights a significant unmet need in cancer care in LMICS, owing to factors such as insufficient healthcare resources, limited access to early detection and treatment and a scarcity of specialised healthcare personnel. As a result, these countries have higher mortality rates and poorer cancer outcomes.^9^ The economic impact in LMICs is also significant, with rising healthcare costs and a loss of productivity.^10^ Addressing these challenges necessitates interventions that are tailored to the unique socioeconomic and healthcare contexts of LMICs.^10,11^

A pivot towards research focusing on quality improvement and the implementation of cancer control in LMICs is essential for better clinical outcomes.^4^ The repercussions of the COVID-19 pandemic brought to light pronounced health disparities globally, with LMICs grappling with pronounced challenges in their healthcare responses.^2,4^ Cancer is now identified as a primary contributor to global morbidity and mortality, with projections indicating a substantial rise in cases over the forthcoming two decades. Regrettably, LMICs bear a disproportionate share of global cancer incidents.^11^ The escalating cancer burden, coupled with unequal distribution of oncological resources in LMICs, underscores the urgent need to reassess the structural imbalances causing such disparities. Recognising these gaps, several global organisations are now collaborating with LMICs to devise apt solutions. Concurrent innovations, such as point-of-care diagnostics, telemedicine and digitised oncological data collection, herald a new era for cancer care in these regions.^2,4,12,13^ Thus, the urgency for a revitalised vision for healthcare has never been clearer. Digital health tools, inclusive of information technology, can revolutionise service delivery, enhancing public engagement and strengthening frontline medical workers.^3,4^ Telemedical platforms can optimise patient care, reducing the reliance on physical consultations and streamlining the utilisation of medical staff.^4,7,14^ Empowered frontline workers with digital tools can help relieve the strains on primary healthcare systems.^3^

In the field of oncology, e-health technologies cover various applications such as decision support, patient management, treatment planning and big data analytics. These tools play a crucial role in improving the accuracy and effectiveness of cancer care, showcasing the wide-ranging influence of e-health in the field of oncology. For instance, IBM Watson for Oncology, utilise artificial intelligence to provide individualised treatment choices.^15^ Additionally, OncoEMR offers specialised Electronic Medical Record (EMR) systems for the management of cancer care.^16^ Varian Medical Systems combines cutting-edge radiation with imaging and treatment planning software. My Cancer Genome is an internet-based repository that provides comprehensive information on many types of cancer and specific genetic abnormalities associated with focused therapeutic approaches. CancerLinQ, an effort led by the American Society of Clinical Oncology, utilises big data to improve patient outcomes through the analysis of real-world cancer care data.^16–18^

During the pandemic, the vulnerabilities of cancer patients were starkly evident due to their heightened risks.^19,20^ e-health tools have become critical in oncology, aiding patient monitoring, evaluations and modifications in treatment.^21,22^ In resource-rich settings, telemedicine has effectively addressed care delivery gaps, improving patient satisfaction.^23^ However, the unrealised potential of these tools in middle-income countries (MICs), especially during the pandemic, underlines the pressing need for fresh healthcare strategies.^3,4^

Acknowledging the existing literature gaps and the need for context-specific solutions, this study focuses on proposals that are feasible and effective locally and regionally, but with the scope for global influence in cancer management. In this light, our research delves into the intricacies of cancer care in MICs, using Jordan as a benchmark. This study aims to provide insights that could serve not just for Jordan but as a guide for similar healthcare environments worldwide, underscoring the research's distinctive importance. The objectives of this research are: (a) To conduct a situational analysis of the current cancer care model using semi-structured interviews with a multidisciplinary team of oncology care providers. (b) To explore the promise of strategic reforms and e-health technologies in enhancing and reinforcing cancer care service delivery.

## Participants and methods

### Ethical considerations

Ethical approval was obtained from Kingston University's ethical principles for scientific research (approval number/1416).

### Study setting and context

Jordan, centrally situated in the Middle East, offers a distinctive lens for healthcare evaluation. With a population of 10.2 million 47% of whom are women urban living dominates, with 90.3% residing in city areas. Moreover, digital engagement is pronounced, as 92% of Jordanians possess a smartphone.^24,25^ Melhem et al.^7^ have identified digital health literacy (DHL) as an essential and independent predictor for the digital transformation in healthcare among cancer survivors. Their research aligns with the World Health Organisation's (WHO's) emphasis on DHL as a fundamental component for the adoption and utilisation of e-health technologies. Specifically, their study highlights the significance of DHL in enabling breast and colorectal cancer survivors to effectively manage their care through digital platforms.

Historically, Jordan's healthcare system earned praise for exemplary achievements in domains like life expectancy and maternal mortality, outcomes attributable to strategic investments, comprehensive immunisation campaigns and effective primary healthcare implementation.^26^ However, contemporary challenges have arisen, chiefly the significant intake of Syrian refugees which has stretched the healthcare infrastructure.^25^ Additionally, the escalating prevalence of noncommunicable diseases, particularly cancer, necessitates an adaptive reorientation in healthcare strategies.^27^ Jordan's healthcare system comprises a diverse array of service providers, ranging from private hospitals, public and semi-governmental or university hospitals governed by the Ministry of Health (MoH) to specialised facilities such as the King Hussein Cancer Centre (KHCC). Established in 1997, KHCC occupies a unique position as the sole centre in the Middle East dedicated exclusively to cancer care for both adults and children. Functioning as a non-governmental, not-for-profit organisation, KHCC has 148 beds and admits over 2300 new patients each year.^26^ Following the World Bank's recent reclassification, Jordan’s classification shifted from an upper MIC to a lower upper MIC.^28^

Jordan, serving as the study's benchmark, presents a unique healthcare landscape in oncology, characterised by a hybrid model of cancer care delivery. The KHCC exemplifies integrative cancer care, offering state-of-the-art, comprehensive services and multidisciplinary treatment under one roof. In contrast, other cancer care pathways in Jordan are delivered through multiple institutions, often leading to less mature cancer units and fragmented treatment trajectories. Additionally, Jordan's position as a stable centre amidst a region marked by conflict and geopolitical turbulence has established it as a regional healthcare hub, attracting patients from neighbouring countries. This duality in its healthcare system, reflecting characteristics of both HICs and MICs, makes Jordan an ideal context to investigate the potential of digital health reforms. Such e-reforms aim to enhance system resilience and optimise healthcare delivery in oncology settings, bridging the gap between various models of care and addressing the challenges posed by the geopolitical landscape. For instance, Jordan's recent downgrade by the World Bank to a lower MIC underscores its economic challenges and the government's constrained ability to subsidise healthcare projects and infrastructure. This shift necessitates exploring novel, feasible methods to bolster the healthcare system. Emphasising the potential of e-health technologies offers a strategic avenue to enhance healthcare delivery within these economic constraints.^28^

### Study design, recruitment and sampling procedure

This exploratory study is anchored in a realism paradigm, asserting the existence of a distinct, independent reality examined through empirical methods within complex social scenarios. The study emphasises theoretical frameworks while acknowledging the interpretative role of the researcher, considering the influences of social, cultural and historical contexts on the reality being studied.^29,30^ Recruitment for the study occurred in two stages. Initially, the first author (SJM) purposively approached prominent oncologists, an oncology pharmacist, nursing experts and a clinical nurse specialist to ensure a diverse range of experienced voices.^31^ Unfortunately, one oncologist opted out. The subsequent phase utilised snowball sampling. Existing participants were prompted to refer other qualified HCPs from their networks, broadening the participant base and ensuring comprehensive representation.^32,33^

The study employed in-depth interviews to understand the perspectives of a multidisciplinary panel of oncology healthcare providers across all sectors of the Jordanian health landscape. A series of semi-structured interviews were conducted with oncology HCPs providers. Potential participants were informed about the study's objectives through a participant information sheet (PIS), which was disseminated via email, WhatsApp, or in-person. Depending on the participants’ preferences, interviews were conducted either in-person or through online platforms such as Skype, Zoom, Facetime and Google Meet. In-person interviewees provided written consent, while those engaged virtually offered verbal consent. All consents were diligently documented in a master list, along with the details of PIS distribution (provided in Supplemental material 1).

Spanning from July 2021 to December 2022, this study was the first phase of a two-part project centred on the potential of digital health in refining healthcare delivery. While the current research delved into the challenges and opportunities of digital health, the next phase explored the wider spectrum of barriers, facilitators, and strategies for the adoption and sustainability of digital health solutions in cancer care within MICs. Findings from the latter are presented in an upcoming separate publication. The first author (SJM) conducted interviews with HCPs, using both in-person and virtual methods, with the conversations conducted in Jordanian Arabic. These interactions were transcribed verbatim and subsequently translated into English by SJM. An anonymisation process was then undertaken. To ensure thorough validation, the co-author (SNG) and (RK), proficient in both English and Arabic, carefully reviewed a subset of the English transcripts for linguistic accuracy and reliability.

### Data collection

Data collection was carried out using semi-structured interviews. The interview topic guide was specifically developed to perform a situational analysis of the prevailing challenges and healthcare delivery models for cancer care. Moreover, it aimed to explore the potential opportunities for integrating digital health solutions as tools for digital reforms within the context of cancer care in the healthcare system. The interview topic guide (Supplemental material 2), rooted in an extensive review of literature on e-health solutions and the intricacies of cancer care within LMICs from the perspective of oncology HCPs,^34–40^ was segmented into eight sections. Prior to deployment, the guide underwent pilot testing with a fellow clinical pharmacist to ensure its clarity and the appropriateness of question formulation. It was designed not only to guide but also to allow flexible and comprehensive examination of distinct topics. Participants were free to skip questions that did not pertain to their specific expertise.

### Data analysis, reporting and rigour

Braun and Clarke's^41^ inductive thematic analysis served as the foundation for our analytical approach. The process commenced with thorough familiarisation with the data, where the first author (SJM) immersed herself in the transcripts repeatedly. The subsequent stages involved actively identifying relevant data excerpts, coding them and methodically grouping these codes into initial themes. Each theme was rigorously defined and continuously refined, ensuring their relevance and alignment with the dataset. This approach acknowledges the researcher's active role in shaping themes, rather than passively allowing them to emerge.

The first author (SJM) initiated the coding process, creating a preliminary list of codes. This coding frame was then meticulously scrutinised and collaboratively refined by co-authors (SNG and RK). For efficient data management and coding, NVivo 12 software was utilised, allowing for the dynamic adjustment of the coding structure as analysis progressed. Our approach to data analysis was guided by the principle of thematic development,^42^ which underscores the researcher's interpretive role in comprehensively exploring research questions and actively developing themes. To ensure thoroughness and validity, the principle of theoretical saturation was pursued, with additional three interviews analysed post-saturation for confirmation of findings.

In maintaining an ethical and balanced engagement with participants, the research team ensured the authenticity and unbiased nature of the study. Transparency in communicating the study's objectives and affiliations was maintained and participant quotes were judiciously used to support thematic interpretations. In the results section, participant quotes are formatted as: (HCP number, specialty, healthcare sector, gender, age). Interview participation was optional, and healthcare professionals received no compensation for their involvement.

To uphold quality and rigour, our reporting of the qualitative findings adheres to the consolidated standards for reporting qualitative research (COREQ).^43^

## Results

### Participants

In the study, 22 oncology specialists underwent interviews, each averaging 80 min, with durations ranging between 53 and 127 min. Table 1 details the participants’ demographics. The cohort included 9 consultant oncologists, with a male to female ratio of 6:3. Among these, one served as a senior administrator in a university hospital, and another at KHCC. The study also encompassed one male senior oncology fellow, four resident doctors (three males and one female), two female clinical nurse specialists, three registered practical nurses (with two males) and three female board-certified oncology pharmacists. All participants operated within Amman, Jordan's capital.

**Table 1.** Demographics of study participants (N = 22).

Variables	N (%)
Age (years)	Range (29–69), median = 42
Sex	
Male	12 (54.5)
Female	10 (45.5)
Role/position	
Oncologist/Consultant (surgical/medical/radiologist)^a^	9 (40.9)
Senior Fellow/Resident Physician (Junior/fellow)	6 (27.2)
Clinical Nurse Specialist (CNS)	2 (9.1)
Registered Nurse	3 (13.6)
Oncology Pharmacist/PharmD	3 (13.6)
Medical Practice Setting	
Cancer Centre	5 (22.8)
University Hospitals	6 (27.2)
Public Sector	5 (22.8)
Private Sector	4 (18.1)
Military Services	2 (9.1)
Working Experience in Oncology (years)	Range (5–38), median = 13

^a^ Consultant Oncologists, encompassing surgical, medical and radiation specialties. In this study, of the nine consultants, six were males—with one a senior manager at a university hospital and another at KHCC—and three were females.

**Figure 1.**
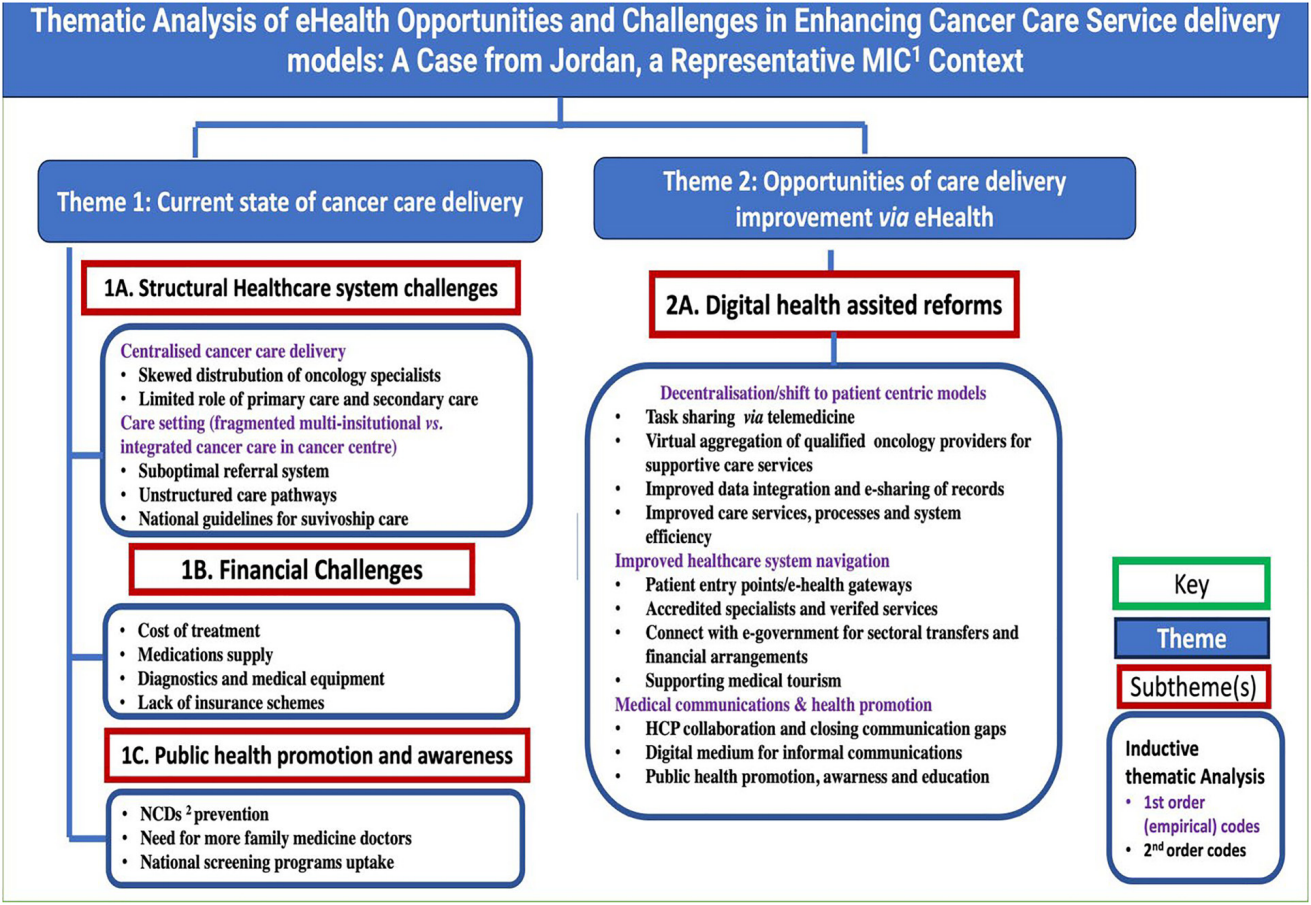
Thematic analysis of e-health opportunities and challenges in enhancing cancer care service delivery in Jordan.

Thematic analysis, as depicted in Figure 1, revealed two overarching themes

#### Theme 1: current state of cancer care delivery

##### Structural health care challenges

The current paradigm for cancer care delivery in Jordan is centralised, with all stages of care, from diagnosis to follow-up, being provided at tertiary care hospitals or the cancer centre KHCC, with minimal involvement and input from primary care institutions. This unstructured care delivery model is driven by the absence of insurance systems and government centralised subsidies for cancer care with the high treatment costs. Additionally, there is uneven distribution of oncology specialists and the high demand for specialised care requiring highly qualified oncology care professionals. A lack of training and development to integrate primary and secondary care for effective follow-up care is a major challenge. This is exacerbated by the fact that primary and secondary care clinicians lack the credentials and expertise to participate in some care activities.

We have a restricted role for primary care for cancer care in Jordan. (HCP3, medical oncologist, private sector, female, 57 years)

Cancer care is expensive, requires comprehensive and collaborative care, integrated activities, and high levels of coordination between medical teams, and it goes beyond merely giving patients chemotherapy or radiotherapy and access to care facilities. (HCP1, surgical oncologist, university hospital director, semi-governmental, male, 69 years)

Family medicine helps manage other chronic diseases, but not cancer. Cancer patients refuse to see a GP, they want their oncologist even for simple issues …. we can’t blame them because there is no integrated care …. Private insurers avoid long, expensive treatments like cancer and mental health, so the government covers these costs. (HCP2, senior oncology manager, KHCC, semi-governmental, female, 56 years)

General doctors can’t provide follow-up for cancer patients in the current setting because there should be a training programme and collaboration with specialists … also many patients are fussy and distrust primary care doctors or even resident doctors, they only want to reach out for the oncologists who carry the brunt of everything especially during treatment. (HCP3, medical oncologist, private sector, female, 57 years)

Medical professionals providing oncology care must be highly specialised, which is challenging to do outside of specialised cancer hospitals or other oncology-based institutions, which is why they require continuous training. (HCP14, CNS, KHCC, semi-governmental, female, 34 years)

The centralisation of care delivery means that services are organised around the system (system-centric), limiting patient access to treatment and autonomy, as it confines the patient to the physical boundaries of the hospital where they are treated. Consequently, patients who live in remote areas must make long journeys to reach the hospital that has been authorised to care for them, even if it is only to receive laboratory services such as blood testing, x-rays, the administration of intravenous drugs and other laboratory services. The centralisation of these services and their confinement to a specific area, augmented by the lack of shared electronic health records and places strain on tertiary hospitals, as many patients come solely for non-clinical appointments, such as prescription renewals, lab work and other such services.

Patients pay a little fraction of the price of their treatment, they come to dispense medications or get their labs from the hospital where they are registered, sometimes their drugs can’t be found in other hospitals near them, as there is no shared electronic system …. (HCP1, surgical oncologist, university hospital director, semi-governmental, male, 69 years)

The absence of inter-organisational exchange of patient data and medical records further hinders treatment decentralisation, as each sector has its own EHR/EMR standards. Patients can only obtain written treatment summaries because there is no portal system in the current model. The patient's treatment plan is carried out in a multi-institutional setting, where the patient must physically navigate several treatment facilities to complete multi-modality care, rather than in a comprehensive care setting in the cancer centre, where treatment is integrated from start to finish. The lack of interoperability restricts collaboration between multiple care providers.

In many cases, doctors in various institutions don’t contact one another unless the patient does this work to get them to discuss the patient's case and plan. This happens when the patient takes their medical report to another facility to continue treatment. (HCP4, haematologist-oncologist, military services, male, 47 years)

In the current practise, we interact with other specialists by phone, especially if we know the specialist or if the patient desires that, and sometimes we just depend on the medical report and fulfil the plan, but I feel regardless that inter-specialist communications have an impact on the patients’ outcomes, I believe it will improve patients’ quality of care and comfort, if we can connect everyone involved in the communication loops. (HCP4, haematologist-oncologist, military services, male, 47 years)

The unstructured healthcare pathways, inadequate referral system and random entry points to the treatment journey exacerbate these problems. Because of the lack of structure, HCPs state that Jordanians rely heavily on informal referral channels (e.g., family and friends) rather than formal ones when seeking medical treatment. They also may seek private consultations and treatment at start to avoid delays.

Our referral system is inadequate, so patients who walk in to the ER or go straight to the outpatient clinics will be seen and treated swiftly. (HCP7, medical oncologist, public sector, male, 49 years)

I think the difficulty is between experiencing symptoms and seeing a doctor, which depends on the patient. (HCP1, surgical oncologist, university hospital director, semi-governmental, male, 69 years)

Patients frequently seek advice from someone they trust about where to go. (HCP11, oncology pharmacist, private sector, female, 37 years)

Getting a private provider is similar to going to the market. I acknowledge that we have excellent providers, but some patients don’t understand that the cancer journey is a long one that requires multiple modalities, and time to seek care is crucial. Some patients just want to start, so they go to a private facility and get surgery. When they become financially strapped, they are transferred to the public sector. How quickly you are transferred depends on the strength of your wasta (favouritism through personal connections) and if you know what to do. (HCP12, oncology pharmacist, private sector, female, 36 years)

##### Financial challenges

Due to the high cost of cancer treatment in Jordan, private insurers do not cover cancer care, and thus there are no insurance programmes, making it hard for many cancer patients to afford treatment and thus they rely on payment exemption, subsidised by governmental funding.

Despite the fact that 90 percent of patients do not have the financial resources to cover their care, Jordan is at the forefront of cancer care by providing free therapy to all residents. (HCP6, surgical oncologist, military services, male, 63 years)

Cancer patients are always given first priority in receiving treatment exemptions from the Prime Ministry or the Royal Court. (HCP7, medical oncologist, public sector, male, 49 years)

The government's emphasis is on paying treatment expenses, with less funding dedicated towards enhancing other areas of care. Due to budgetary constraints, such a focus diverts funding away from initiatives to improve medical infrastructure, train and hire more trained professionals, invest in screening methods, improve supportive care services and genetic testing and provide more modern diagnostic tools such as positron emission tomography (PET) scans, robots and technology-enabled care. The high cost of medicine delivery, especially antineoplastics and diagnostics, further limits resources for these advancements. Participants said the sector relies on contributions and cooperation from foreign health organisations to expand cancer care and overcome budgetary restrictions.

The ministry of health refers many patients to us, but when we inform them of the bill—let's say 30 million JD—they begin to negotiate, and as a result, they only pay us 10 million JD for example. As a result, we have little capital for innovation because our budget goes towards meeting basic needs. (HCP1, surgical oncologist, university hospital director, semi-governmental, male, 69 years)

##### Public health promotion and awareness

Existing cancer screening, treatment and prevention practises were deemed insufficient and ineffective. Cancer professionals stressed the importance of NCD prevention in general and educating the public about lifestyle changes, behavioural changes and chronic disease prevention, including cancer. This requires funding and effective primary care structure. One major issue is Jordanians’ high hookah (water pipe or shisha) and cigarette smoking rates. Additionally, the Mediterranean diet has been replaced by fast and processed food, making risk factor control and modification more important. There needs to be a change of public and governmental prioritisation and expenditure towards prevention.

NCDs prevention should be better, we need to address the root cause of the problem … perhaps through good primary care facilities … the public needs to be informed of the services available and how they can easily access them … if things are for prevention many people don’t care. So, we to make it very accessible services … . currently the expenditures are just like an inverted pyramid …. (HCP2, senior Oncology manager, KHCC, semi-governmental, female, 56 years)

They [policymakers] recognise this is a significant problem [smoking], but if you decide to stop, for example, you’ll quit your quitting choice because they don’t know where to go to get patches or nicotine gums, they should be for free … Without actual change, government officials are essentially blowing hot air. (HCP2, senior Oncology manager, KHCC, semi-governmental, female, 56 years)

We [Jordanians] spend more money on tobacco than food …. (HCP16, nurse, public sector, semi-governmental, male, 39 years)

This is a ticking time bomb; we have one of the highest smoking rates in the world. (HCP10, senior fellow oncologist, KHCC, semi-governmental, male, 40 years)

The role of the family physician in the screening, prevention and ongoing care of cancer patients is crucial. Through risk assessments, they can aid in identifying those at high risk for cancer, suggest screening tests and counsel on alterations to one's lifestyle to reduce cancer risks. Further, family physicians can treat and support mental health during treatment and survivorship. Better communication between primary care physicians and oncologists is needed to provide patients with comprehensive cancer care. Integrating family medicine and oncology services can improve patient outcomes and reduce cancer incidence across the country by allowing for more comprehensive and coordinated cancer care to be provided. However, psychiatrists, family doctors and primary care physicians are often undervalued. Oncologists believe family medicine and prevention should be strengthened and empowered to address rising cancer rates due to staff shortages and public scepticism.

Family medicine and mental health specialties are the most important; they are constantly marginalised by society due to unintentional ignorance of their importance, or other specialists and policy makers look down on them incorrectly, so their role is marginalised to satisfy the interests of certain players in the healthcare system … what is the point as a society if you have excellent specialists and the cases of cancer and chronic diseases continue to rise?!!! or if you have a state-of-the-art cancer centre and spend millions on cancer care if you are deficient in providing access to cancer screenings like colonoscopies ….. this can’t be done without expanding the role of family practitioners to screen the family. (HCP3, medical oncologist, private sector, female, 57 years)

#### Theme 2: opportunities of care delivery improvement via e-health

##### Digital health assisted reforms

Oncologists said task sharing *via* telemedicine, when experts delegate certain tasks to primary care or family medicine practitioners, can be beneficial. Specialists and primary care doctors can collaborate to provide comprehensive cancer care, improving patient outcomes. In the follow-up phase of cancer survivors, primary care clinicians can help with screening for cancer recurrence, managing treatment-related side effects and providing psychosocial support to improve patient satisfaction.

Telemedicine was also deemed to allow virtual aggregation of qualified cancer providers for supportive care services. This method is particularly useful in locations with a shortage of specialised oncology providers or supportive care services that are frequently inadequate due to disproportionate availability of cancer care providers. Participants indicated that patients can obtain low-cost electronic services such as psychosocial assistance, medication counselling and nutritional support, hence boosting access to nearby care providers. The ability to electronically being connected with oncology providers compensates for the absence of expertise in certain treatment facilities, allowing patients access to these services regardless of location. Furthermore, this model of care is reassuring as would preserve patients’ trust because they would still be supervised by their treating physician.

Our hospital is not a dedicated cancer centre, so while we do have a multidisciplinary panel to help with patient treatment planning, we may benefit from being connected with clinical nurse specialists, oncology pharmacists, and oncology radiologists. This is because, unlike at a cancer centre, oncologists in public, private, and academic medical centres often lack the resources of a fully integrated cancer care team. (HCP12, oncology pharmacist, private sector, female, 36 years)

… there is an uneven distribution of specialised personnel among the care facilities, such as the presence or absence of social workers in various locations. So, maybe if we had a platform that let people share their knowledge and bring them together virtually, they could help with counselling, education, or other non-clinical interventions that could be done from a distance. (HCP14, CNS, KHCC, semi-governmental, female, 34 years)

As a result, most HCPs believe a national telemedicine programme to be a priority in shifting to patient-centric models that employ digital health to increase access to care resources within close proximity to patients, alleviating the strain on tertiary care and large hospitals.

To incorporate e-consultations, then we absolutely must have a national telemedicine programme in place to back up and supplement any electronic platform or mobile app for patients. (HCP1, surgical oncologist, university hospital director, semi-governmental, male, 69 years)

A comprehensive e-health portal system or database approved by the MoH or other regulating organisations could assist in closing existing gaps in the healthcare system. Patients typically have trouble navigating the complex and unstructured healthcare system, which delays care. Patients could use the comprehensive portal system to better navigate the system and get preventive and follow-up care. One method of accomplishing this is through making use of a person's national insurance number and other personal information available on the government's website. This information could be used to streamline cancer care service portfolios and support screening initiatives by providing booking services, facilitating private-public sector resource allocation, accommodating screening services and creating virtual spaces for organising and allocating services to meet patients’ needs. Further, this e-gateway may allow patients to manage electronic transfers depending on insurance type in addition to the vital information about specialists.

The patient e-gateway portal system may improve patient navigation and care access by simplifying electronic transfers, improving the flow of information between administrative divisions and offering information on the care pathway. For instance, patients with chronic conditions like diabetes or cardiovascular disease could be steered through the healthcare system to find relevant resources and treatments. This would aid patients with communication, and geographically related access issues. It is crucial for patients to be able to choose the right provider with the proper credentials. One participant has noted that the provision of such a portal will allow for a greater enforcement of regulations by including only accredited providers with relevant experience and credentials, especially those working in the private sector.

I believe the portal can simplify healthcare processes and how patients should begin and track down their journey based on their insurance type. Therefore, it would be a major leap for the healthcare sector, if a national electronic health gateway system is implemented, and the government should be involved for financial matters. In this case, patients can have full access to their treatment route, who are their caring providers, and their contact details. (HCP1, surgical oncologist, university hospital director, semi-governmental, male, 69 years)

Rarely do we witness this in cancer care, as patients, once aware of their diagnosis, form bonds with their care teams. Yet, I can envision the value in a portal populated with verified details of specialists, encompassing their contact information and areas of expertise. This could be authenticated by esteemed bodies such as the medical association or the Ministry of Health, adding a layer of trust …. Such portals could have an impactful role if they include liaison physicians tasked with elucidating the patients’ or their families’ medical conditions in general, explain early consultations and providing directions ….. This would be particularly useful for those seeking multiple opinions, navigating through the maze of ‘doctor shopping’ and unfamiliar with the concept of multidisciplinary care. (HCP3, medical oncologist, private sector, female, 57 years)

Also, given that Jordan is home to the only comprehensive cancer centre in the Middle East, this e-gateway may prove useful in attracting international patients in need of cancer treatment to Jordan.

If there are virtual clinics or safe digital channels, it can also help us keep connected with patients who come here from nearby countries primarily for treatment, which I believe may be a significant additional benefit …. (HCP3, medical oncologist, private sector, female, 57 years)

## Discussion

### Current state of cancer delivery

The provision of cancer care in MIC such as Jordan confronts significant structural, financial and public health challenges that must be addressed in order to improve patient outcomes and reduce cancer incidence. Addressing these challenges will require a multifaceted strategy. This strategy includes improving medical infrastructure, training and hiring more qualified professionals, investing in screening methods, enhancing supportive care services, promoting healthy behaviours and reducing cancer risk factors through public health campaigns, and integrating family medicine and oncology services to improve patient outcomes and decrease the incidence of cancer.^44,45^

A centralised healthcare system in Jordan limits patient autonomy and access to care, as patients must travel considerable distances to the approved hospital to treat them. Due to the lack of cancer insurance and government subsidies, treatment costs are high and tertiary hospitals are overburdened. The lack of training and development to integrate primary and secondary care professionals into follow-up care is another key barrier to their involvement in cancer care. These findings are consistent with prior research on cancer care delivery in LMICs countries, which has stressed the necessity of resolving structural health care barriers to enhance patient outcomes and reduce cancer incidence.^46^

Private insurers do not cover cancer care in Jordan, and there are no insurance initiatives to help patients finance treatment. The government prioritises capacity building in oncology health services including prioritising treatment costs above upgrading medical infrastructure, training and hiring more trained experts, screening measures and supporting care. These problems are also consistent with past research on cancer care finance in LMICs, which has highlighted the need for novel financial strategies to increase access to cancer care.^47^

As existing cancer screening, treatment and prevention practises were deemed suboptimal, cancer care delivery in Jordan also requires a focus on public health promotion and awareness. Professionals in the field of cancer highlighted the significance of NCD prevention in general and educating the public about lifestyle changes, behavioural changes and chronic illness prevention, including cancer. High rates of smoking among Jordanians, particularly hookah and cigarettes, are an alarming problem that highlights the significance of encouraging healthy behaviours and lowering cancer risk factors through public health initiatives. These findings are consistent with prior research on the role of cancer prevention and early detection in reducing cancer mortality.^48,49^

In this context, historically, LMICs have struggled to reduce cancer risk variables. This is due to a variety of factors, including cultural barriers and insufficient infrastructure. Hence, understanding and overcoming these barriers could improve risk factor modification in LMICs. The results only partly support previous research suggesting that early identification *via* screening and surveillance, which is practised in HICs, may not yield the same benefits in LMICs, where limited downstream treatment resources may struggle to manage the anticipated increase in cancer diagnoses. To avoid unanticipated results, cancer control programmes must be well-balanced.^50^

Family physicians are marginalised despite their potential to play a crucial role in the delivery of cancer care, as they can identify individuals at high risk for the disease, recommend screening exams, offer advice on lifestyle modifications and provide mental health support throughout treatment and survivorship. Integrating general medicine and oncology services can enhance patient outcomes and reduce cancer incidence nationwide by providing more comprehensive and coordinated cancer care. These results are in line with earlier studies in HICs that have highlighted the value of primary care in cancer prevention, early detection and survivorship care.^51–53^

### Opportunities of care delivery improvement *via* e-health

The second theme showed that digital health technologies, particularly telemedicine, offer significant opportunities for improving cancer care delivery in MICs, highlighting the need to identify the best strategies for implementing telemedicine and e-health portals in cancer care to improve access to care resources and patient outcomes^54,55^ .Telemedicine's ability to task sharing between oncology experts and general care physicians can enhance patient outcomes by delivering comprehensive cancer care, particularly in cancer survivors’ follow-up^54,55^ .Virtually aggregating qualified cancer providers for supportive care can address the shortage of specialised oncology providers, especially in areas with limited services. Patients can use low-cost electronic services such as psychosocial support, medication counselling and nutritional help, enhancing access to alternative care providers.^56^ A comprehensive e-health portal system or database approved by the MoH or other regulatory organisations could help to close existing gaps in the healthcare system, particularly in patient navigation and service access through virtual structuring of healthcare sector. The digitisation of healthcare services is a transformative trend that places immense importance on the quality of healthcare websites. Numerous studies offer comprehensive frameworks to evaluate such websites based on readability, accessibility, content quality and user orientation.^57^ These benchmarks are not just vital for healthcare in a traditional sense but are also integral to the growing field of telemedicine, particularly in specialised care sectors such as cancer care in MICs.^54,55^ Telemedicine represents a revolutionary avenue in healthcare delivery, especially in niche areas like oncology. It allows for effective task-sharing between expert oncologists and general physicians, leading to improved patient outcomes.^54,55^ In addition to addressing the scarcity of specialised healthcare providers through virtual aggregation, telemedicine platforms can offer affordable auxiliary services such as psychological support, medication counselling and dietary guidance.^56^ However, there are potential challenges that must be addressed when implementing telemedicine and e-health portals in cancer care.

These include issues of patient privacy and the absence of well-defined reimbursement structures, are important impediments to broad use of telemedicine in cancer therapy.^58^ Furthermore, and as outlined in the results of previous chapters, patient Health Literacy (HL), DHL and language barriers may limit the effectiveness of e-health portals and databases.^7,46–48^ Despite these challenges, the findings suggest that a national telemedicine programme is a priority for shifting to patient-centric models that use digital health to increase access to care resources close to patients, thereby relieving the strain on tertiary care and large hospitals.

These findings are consistent with previous studies that have shown that telemedicine can improve patient outcomes, particularly in chronic diseases, by increasing access to care, improving patient self-management and reducing healthcare costs.^54,55^ Telemedicine has been demonstrated to enhance patient outcomes in oncology by giving prompt access to specialist treatment, minimising hospital admissions and increasing patient satisfaction.^59^

Additionally, to improve public health and implement e-health reforms, it is crucial to investigate AI algorithms. Effective strategies should encompass updating data governance, enhancing data and analytic infrastructure, bridging the skills gap in AI and data science, forging strategic partnerships and ensuring transparency and fairness in AI practices. This approach, prioritising equity and bias considerations, is crucial for advancing health outcomes across diverse populations and is fundamental in future research agenda's regarding AI's potential e-reforms in public health information campaigns.^60^

## Limitations and conclusions

In this qualitative study, we comprehensively examined the cancer care delivery models in Jordan's healthcare system, highlighting the role and potential of digital interventions as solution to current challenges. By obtaining insights from oncology care providers in a Middle Eastern MIC, the study's validity was strengthened. However, the specificity of the qualitative methodology and the regional focus on Jordan might limit the generalisability of the findings. Additionally, a noticeable gap was the limited input from the primary care sector.

For future research, it's essential to investigate best practices for expanding digital health initiatives in LMICs. Practical insights from real-world case studies can offer valuable guidance, shaping the development and deployment of health programmes in diverse contexts. In the light of these discussions, telemedicine emerges as a significant player. A key objective remains to effectively integrate digital strategies with traditional care methods, ensuring patient-centricity remains at the heart of evolving healthcare models.

## Supplemental Material

sj-docx-1-dhj-10.1177_20552076241237668 - Supplemental material for Leveraging e-health for enhanced cancer care service models in middle-income contexts: Qualitative insights from oncology care providers Supplemental material, sj-docx-1-dhj-10.1177_20552076241237668 for Leveraging e-health for enhanced cancer care service models in middle-income contexts: Qualitative insights from oncology care providers by Samar J Melhem, Shereen Nabhani-Gebara and Reem Kayyali in DIGITAL HEALTH

sj-docx-2-dhj-10.1177_20552076241237668 - Supplemental material for Leveraging e-health for enhanced cancer care service models in middle-income contexts: Qualitative insights from oncology care providers Supplemental material, sj-docx-2-dhj-10.1177_20552076241237668 for Leveraging e-health for enhanced cancer care service models in middle-income contexts: Qualitative insights from oncology care providers by Samar J Melhem, Shereen Nabhani-Gebara and Reem Kayyali in DIGITAL HEALTH

## References

[1] Ranganathan P Chinnaswamy G Sengar M , et al. The international collaboration for research methods development in oncology (CReDO) workshops: shaping the future of global oncology research. Lancet Oncol 2021; 22: e369–e376. 10.1016/S1470-2045(21)00077-2 PMC8328959 34216541

[2] Grewal US Shankar A Saini D , et al. Tele-health and cancer care in the era of COVID-19: new opportunities in low and middle income countries (LMICs). Cancer Treat Res Commun 2021; 27: 100313. 33465561 10.1016/j.ctarc.2021.100313 PMC7833952

[3] Pramesh CS Badwe RA Bhoo-Pathy N , et al. Priorities for cancer research in low-and middle-income countries: a global perspective. Nat Med 2022; 28: 649–657. 35440716 10.1038/s41591-022-01738-x PMC9108683

[4] Mitgang EA Blaya JA Chopra M . Digital health in response to COVID-19 in low-and middle-income countries: opportunities and challenges. Global Policy 2021; 12: 107–109. 34230840 10.1111/1758-5899.12880 PMC8250781

[5] Frost MJ Tran JB Khatun F , et al. What does it take to be an effective national steward of digital health integration for health systems strengthening in low- and middle-income countries? Global Health: Sci Pract. 2018; 6(Supplement 1): S18–S28. 10.9745/GHSP-D-18-00270 PMC6203416 30305336

[6] McCool J Dobson R Whittaker R , et al. Mobile health (mHealth) in low-and middle-income countries. Annu Rev Public Health 2022; 43: 525–539. 34648368 10.1146/annurev-publhealth-052620-093850

[7] Melhem SJ Nabhani-Gebara S Kayyali R . Digital trends, digital literacy, and e-health engagement predictors of breast and colorectal cancer survivors: a population-based cross-sectional survey. Int J Environ Res Public Health 2023; 20: 1472. 36674237 10.3390/ijerph20021472 PMC9860554

[8] Torre LA Siegel RL Ward EM , et al. Global cancer incidence and mortality rates and trends—an update. Cancer Epidemiol Biomarkers Prev 2016; 25: 16–27. 26667886 10.1158/1055-9965.EPI-15-0578

[9] Drake TM Knight SR Harrison EM , et al. Global inequities in precision medicine and molecular cancer research. Front Oncol 2018; 8: 346. 30234014 10.3389/fonc.2018.00346 PMC6131579

[10] Sivaram S Perkins S He M , et al. Building capacity for global cancer research: existing opportunities and future directions. J Cancer Educ 2021; 36: 5–24. 34273100 10.1007/s13187-021-02043-w PMC8285681

[11] Sommer I Griebler U Mahlknecht P , et al. Socioeconomic inequalities in non-communicable diseases and their risk factors: an overview of systematic reviews. BMC Public Health 2015; 15: 1–2. 26385563 10.1186/s12889-015-2227-y PMC4575459

[12] Haney K Tandon P Divi R , et al. The role of affordable, point-of-care technologies for cancer care in low-and middle-income countries: a review and commentary. IEEE J Transl Eng Health Med 2017; 5: 1–4. 10.1109/JTEHM.2017.2761764 PMC5706528 29204328

[13] Ngwa W Olver I Schmeler KM . The use of health-related technology to reduce the gap between developed and undeveloped regions around the globe. Am Soc Clin Oncol Educ Book 2020; 40: 227–236. 10.1200/EDBK_288613 32223667

[14] Pesec M Sherertz T . Global health from a cancer care perspective. Future Oncol 2015; 11: 2235–2245. 26235185 10.2217/fon.15.142

[15] Jie Z Zhiying Z Li L . A meta-analysis of Watson for oncology in clinical application. Sci Rep 2021; 11: 5792. 33707577 10.1038/s41598-021-84973-5 PMC7952578

[16] Potter D Brothers R Kolacevski A , et al. Development of CancerLinQ, a health information learning platform from multiple electronic health record systems to support improved quality of care. JCO Clin Cancer Inform 2020; 4: 929–937. 33104389 10.1200/CCI.20.00064 PMC7608629

[17] Rubinstein SM Warner JL . Cancerlinq: origins, implementation, and future directions. JCO Clin Cancer Inform 2018; 2: 1–7. 10.1200/CCI.17.00060 30652539

[18] Sledge GW Jr Miller RS Hauser R . Cancerlinq and the future of cancer care. Am Soc Clin Oncol Educ Book 2013; 33: 430–434. 10.14694/EdBook_AM.2013.33.430 23714566

[19] Lee LY Cazier JB Angelis V , et al. COVID-19 mortality in patients with cancer on chemotherapy or other anticancer treatments: a prospective cohort study. Lancet 2020; 395: 1919–1926. 32473682 10.1016/S0140-6736(20)31173-9 PMC7255715

[20] Pinato DJ Scotti L Gennari A , et al. Determinants of enhanced vulnerability to coronavirus disease 2019 in UK patients with cancer: a European study. Eur J Cancer 2021; 150: 190–202. 33932726 10.1016/j.ejca.2021.03.035 PMC8023206

[21] Kelley MM Kue J Brophy L , et al. Mobile health applications, cancer survivors and lifestyle modification: an integrative review. Comput Inform Nurs 2021; 39: 755. 34074873 10.1097/CIN.0000000000000781 PMC8578050

[22] Girgis A Durcinoska I Levesque JV , et al. e-health system for collecting and utilizing patient reported outcome measures for personalized treatment and care (PROMPT-care) among cancer patients: mixed methods approach to evaluate feasibility and acceptability. J Med Internet Res 2017; 19: e330. 10.2196/jmir.8360 PMC5667931 28970188

[23] Kendzerska T Zhu DT Gershon AS , et al. The effects of the health system response to the COVID-19 pandemic on chronic disease management: a narrative review. Risk Manag Healthc Policy 2021; 14: 575–584. 33623448 10.2147/RMHP.S293471 PMC7894869

[24] Russell J (ed). Critical issues facing the Middle East: security, politics and economics. Springer, 2006.

[25] Spiegel P Khalifa A Mateen FJ . Cancer in refugees in Jordan and Syria between 2009 and 2012: challenges and the way forward in humanitarian emergencies. Lancet Oncol 2014; 15: e290–e297. PMID: 24872112. 10.1016/S1470-2045(14)70067-1 24872112

[26] Ajlouni M . Jordan health system profile. EMRO: World Health Organization, 2011.

[27] World Health Organization. High level expert meeting on health priorities in the Eastern Mediterranean Region, 1-2 March 2012: addressing priorities and scaling up action to prevent and control noncommunicable diseases. 2012.

[28] https://data.worldbank.org/?locations=JO-XN (accessed 18 September 2023).

[29] Lawani A . Critical realism: what you should know and how to apply it. Qual Res J 2021; 21: 320–333.

[30] Wiltshire G Ronkainen N . A realist approach to thematic analysis: making sense of qualitative data through experiential, inferential and dispositional themes. J Crit Realism 2021; 20: 159–180.

[31] Gentles SJ Charles C Ploeg J , et al. Sampling in qualitative research: insights from an overview of the methods literature. Qual Rep 2015; 20: 1772–1789.

[32] Naderifar M Goli H Ghaljaie F . Snowball sampling: a purposeful method of sampling in qualitative research. Strides Dev Med Educ 2017; 14. doi:10.5812/sdme.67670

[33] Parker C Scott S Geddes A . Snowball sampling. SAGE Research Methods Foundations. 2019 Sep 9.

[34] Gagnon MP Ngangue P Payne-Gagnon J , et al. m-Health adoption by healthcare professionals: a systematic review. J Am Med Inform Assoc 2016; 23: 212–220. 26078410 10.1093/jamia/ocv052 PMC7814918

[35] Akhlaq A McKinstry B Muhammad KB , et al. Barriers and facilitators to health information exchange in low-and middle-income country settings: a systematic review. Health Policy Plan 2016; 31: 1310–1325. 27185528 10.1093/heapol/czw056

[36] Hopstaken JS Verweij L van Laarhoven CJ , et al. Effect of digital care platforms on quality of care for oncological patients and barriers and facilitators for their implementation: systematic review. J Med Internet Res 2021; 23: e28869. 10.2196/28869 PMC8501408 34559057

[37] Pohlmann S Kunz A Ose D , et al. Digitalizing health services by implementing a personal electronic health record in Germany: qualitative analysis of fundamental prerequisites from the perspective of selected experts. J Med Internet Res 2020; 22: e15102. 10.2196/15102 PMC7016629 32012060

[38] Leigh S Ashall-Payne L . The role of health-care providers in mHealth adoption. Lancet Digit Health 2019; 1: e58–e59. 10.1016/S2589-7500(19)30025-1 33323231

[39] Kessel KA Vogel MME Schmidt-Graf F , et al. Mobile apps in oncology: a survey on health care professionals’ attitude toward telemedicine, mHealth, and oncological apps. J Med Internet Res 2016; 18: e312. PMID: 27884810; PMCID: PMC5146327. 10.2196/jmir.6399 PMC5146327 27884810

[40] Berkowitz CM Zullig LL Koontz BF , et al. Prescribing an app? Oncology providers’ views on mobile health apps for cancer care. JCO Clin Cancer Inform 2017; 1: 1–7. 10.1200/CCI.17.00107 30657404

[41] Braun V Clarke V . Using thematic analysis in psychology. Qual Res Psychol 2006; 3: 77–101.

[42] Hennink M Kaiser BN . Sample sizes for saturation in qualitative research: a systematic review of empirical tests. Soc Sci Med 2021; 292: 114523. 34785096 10.1016/j.socscimed.2021.114523

[43] Tong A Sainsbury P Craig J . Consolidated criteria for reporting qualitative research (COREQ): a 32-item checklist for interviews and focus groups. Int J Qual Health Care 2007; 19: 349–357. Epub 2007 Sep 14. PMID: 17872937. 17872937 10.1093/intqhc/mzm042

[44] Brand NR Qu LG Chao A , et al. Delays and barriers to cancer care in low-and middle-income countries: a systematic review. Oncologist 2019; 24: e1371–e1380. 10.1634/theoncologist.2019-0057 PMC6975966 31387949

[45] Donkor A Luckett T Aranda S , et al. Barriers and facilitators to implementation of cancer treatment and palliative care strategies in low-and middle-income countries: systematic review. Int J Public Health 2018; 63: 1047–1057. 29974131 10.1007/s00038-018-1142-2

[46] Sirohi B Chalkidou K Pramesh CS , et al. Developing institutions for cancer care in low-income and middle-income countries: from cancer units to comprehensive cancer centres. Lancet Oncol 2018; 19: e395–e406. 10.1016/S1470-2045(18)30342-5 30102234

[47] Sullivan R Alatise OI Anderson BO , et al. Global cancer surgery: delivering safe, affordable, and timely cancer surgery. Lancet Oncol 2015; 16: 1193–1224. 26427363 10.1016/S1470-2045(15)00223-5

[48] Abdel-Razeq H Attiga F Mansour A . Cancer care in Jordan. Hematol Oncol Stem Cell Ther 2015; 8: 64–70. Epub 2015 Feb 21. PMID: 25732671. 25732671 10.1016/j.hemonc.2015.02.001

[49] Danaei G Vander Hoorn S Lopez AD , et al. Causes of cancer in the world: comparative risk assessment of nine behavioural and environmental risk factors. Lancet 2005; 366: 1784–1793. PMID: 16298215. 16298215 10.1016/S0140-6736(05)67725-2

[50] Shah SC Kayamba V Peek RM Jr , et al. Cancer control in low-and middle-income countries: is it time to consider screening? J Glob Oncol 2019; 5: 1–8. 10.1200/JGO.18.00200 PMC6452918 30908147

[51] Price ST Berini C Seehusen D , et al. Cancer survivorship training in family medicine residency programs. J Cancer Surviv 2021; 15: 748–754. 33175993 10.1007/s11764-020-00966-9

[52] O’Brien MA Grunfeld E Sussman J , et al. Views of family physicians about survivorship care plans to provide breast cancer follow-up care: exploration of results from a randomized controlled trial. Curr Oncol 2015; 22: 252–259. 26300663 10.3747/co.22.2368 PMC4530810

[53] Haq R Heus L Baker NA , et al. Designing a multifaceted survivorship care plan to meet the information and communication needs of breast cancer patients and their family physicians: results of a qualitative pilot study. BMC Med Inform Decis Mak 2013; 13: 1–3. 23883430 10.1186/1472-6947-13-76 PMC3733735

[54] Al-Khaled T Cole ED Chan RP . Telemedicine, telementoring, and technology: improving patient outcomes and access to care in low and middle-income countries. Ophthalmology 2021; 128: 138–139. 33349339 10.1016/j.ophtha.2020.10.014

[55] Yadav K Ginsburg O Basu P , et al. Telemedicine and cancer care in low- and middle-income countries during the SARS-CoV-2 pandemic. JCO Glob Oncol 2021; 7: 1633–1638. PMID: 34860567; PMCID: PMC8654432. 34860567 10.1200/GO.21.00249 PMC8654432

[56] Hjelm NM . Benefits and drawbacks of telemedicine. J Telemed Telecare 2005; 11: 60–70. 15829049 10.1258/1357633053499886

[57] Sarantis D Soares DS . From a literature review to a conceptual framework for health sector websites’ assessment. In: Electronic government: 16th IFIP WG 8.5 international conference, EGOV 2017, St. Petersburg, Russia, September 4–7 2017, Proceedings 16 2017. Springer International Publishing, pp.128–141.

[58] Hwei LR Octavius GS . Potential advantages and disadvantages of telemedicine: a literature review from the perspectives of patients, medical personnel, and hospitals. J Community Empowerment Health 2021; 4: 228–234.

[59] Sirintrapun SJ Lopez AM . Telemedicine in cancer care. Am Soc Clin Oncol Educ Book 2018; 38: 540–545. 30231354 10.1200/EDBK_200141

[60] Fisher S Rosella LC . Priorities for successful use of artificial intelligence by public health organizations: a literature review. BMC Public Health 2022; 22: 2146. 36419010 10.1186/s12889-022-14422-z PMC9682716

