# Supplementary material for: Leveraging e-health for enhanced cancer care service models in middle-income contexts: Qualitative insights from oncology care providers
Source: Digit Health. 2024 Mar 13;10:20552076241237668. doi: 10.1177/20552076241237668 (PMC10938624; doi:10.1177/20552076241237668)
Supplement: sj-docx-1-dhj-10.1177_20552076241237668 - Supplemental material for Leveraging e-health for enhanced cancer care service models in middle-income contexts: Qualitative insights from oncology care providers [file sj-docx-1-dhj-10.1177_20552076241237668.docx]

**Kingston University London**

**Supplementary 1: Participant information Sheet (PIS) for healthcare providers**

Phase (1) Determination of User Requirements for a Holistic Digital Platform for Collaborative Patient-Centered Care Models in Oncology

[Date: ……/……/ 2020]

You are being invited to take part in a research study. Before you decide whether or not to take part, it is important for you to understand why the research is being done and what it will involve. Please take time to read the following information sheet carefully before deciding whether or not to participate. If you decide not to take part, there will be no disadvantage to you, and we thank you for your time in considering our project. If after reading this information sheet, you are still unsure or uncertain about anything, then we are happy to answer any questions you may have.

What is the purpose of the study?

The primary aim of this study is:

To elicit the experiences and challenges breast and colorectal cancer patients encounter throughout their cancer journey from diagnosis to follow-up. In addition, the research seeks to understand and determine the specific requirements of a digital platform (a mobile app and/or a website) from the users’ perspective. The digital solution will serve cancer supportive care of cancer survivors across the cancer trajectory.

Why have I been chosen?

You are an oncologist who works closely with breast/colorectal cancer patients. Hence, you are in the best position to help us in recruiting eligible cancer survivors to participate in the first phase of the research project. This phase aims to determine the specific user requirements of people living with and beyond cancer (PLWBC) and their informal caregivers through conducting focus groups. The research findings will subsequently guide the design and development of a holistic digital platform to support (PLWBC) from diagnosis to follow-up in Jordan and other Arabic-speaking communities.

The indirect recruitment of subjects is the most feasible and practical way to approach the patients and their informal caregivers who come for follow-up visits at your clinic. The recruitment of subjects can be delegated to a resident physician or a nurse, and the selection of subjects is based on the eligibility criteria in the attached recruitment sheet.

The recruitment process will be carried out through telephone as part of COVID-19 protective measures to limit the potential to cause harm to participants that increase the risk of infection with SARS-CoV-2.

Do I have to take part?

No. This research study is done purely on a voluntary basis. If you choose to take part after reading this information sheet, we will ask you to sign a consent form. You are free to withdraw from this study at any point without disadvantage and without having to provide a reason.

What will happen to me if I take part?

You will be asked to help us in recruiting subjects to be enrolled in one of the focus groups using the recruitment sheet attached.

What are the possible benefits of taking part?

Your support in helping us to recruit breast and/or colorectal cancer patients is crucial to the success of the research project, because you can effectively approach eligible patients during their routine visit for follow-up and ask them to take part in the study. The accomplishment of this project is essential for a successful development that we believe will benefit many cancer patients in Jordan in the future.

What are the possible disadvantages and risks of taking part?

There are no risks associated with taking part in this study. However, we understand that time is valuable to you. Therefore, we greatly appreciate that you will devote some of your time for this study. The recruitment could be assigned to a member of your medical team and will be performed during routine timings of the appointments to your clinic.

What happens when the research study ends?

You will be under no obligation to volunteer again. Contact details for myself plus project supervisor are included at the end of this information sheet should you wish to discuss the findings.

Will my taking part be kept confidential?

All information collected during the course of the study will be kept strictly confidential and in secure storage. Only the main researcher: Samar Melhem and the project supervisor: Prof. Reem Kayyali will have access to this dataset.

Any personal information collected will be immediately destroyed, except that required by the University research policy.

Who is organising and funding the study?

This study is part of a research project, within the School of Life Sciences, Pharmacy and Chemistry at Kingston University. None of the investigators stands to gain financially from this study.

What will happen to the results of the research study?

The results will be made available in the Faculty of Science Learning Resources Centre (library) at Kingston University for others to view. In addition, findings arising from this study may be presented at national and international conferences as well as published in scientific journals. It will not be possible to identify you or others from any such publications with results being aggregated for the whole group.

Please contact me, : Samar Melhem or Prof. Reem Kayyali or Dr. Shereen Nabhani, if you have any questions about this project.

Who has reviewed the study?

The study has been reviewed and approved by Jordan University Hospital Institution Review Board (IRB). In addition, The Kingston University Faculty of Science Research Ethics Committee has reviewed and approved this study.

Also, this project is being supervised by Prof. Reem Kayyali and Dr. Shereen Nabhani-Gebara.

Contact for further information.

Further information may be obtained from:

• Samar Melhem,

E-mail: K1831594@kingston.ac.uk

Tel: 00447732720203

Tel :00962785554464

• Project supervisor: Prof. Reem Kayyali

Faculty of Science, Engineering and Computing, Kingston University London

Penrhyn Road

Kingston upon Thames

Surrey KT1 2EE

E-mail: R.Kayyali@kingston.ac.uk

Tel: +44 (0)20 8417 2561.

• Second supervisor: Dr. Shereen El. Nabhani-Gebara

Faculty of Science, Engineering and Computing, Kingston University London

Penrhyn Road

Kingston upon Thames

Surrey KT1 2EE

E-mail: [s.nabhani@kingston.ac.uk](mailto:s.nabhani@kingston.ac.uk)

If you become concerned about any issue that may have been raised by you participating in this study, please contact Prof. Reem Kayyali on details provided above.
