# Supplementary material for: Leveraging e-health for enhanced cancer care service models in middle-income contexts: Qualitative insights from oncology care providers
Source: Digit Health. 2024 Mar 13;10:20552076241237668. doi: 10.1177/20552076241237668 (PMC10938624; doi:10.1177/20552076241237668)
Supplement: sj-docx-2-dhj-10.1177_20552076241237668 - Supplemental material for Leveraging e-health for enhanced cancer care service models in middle-income contexts: Qualitative insights from oncology care providers [file sj-docx-2-dhj-10.1177_20552076241237668.docx]

**Kingston University London**

**Supplementary 2: Interview topic guide with Oncology care providers**

Interview Topic Guide for Oncology Care Providers at Kingston University London - Integrating E-health Solutions and Cancer Care in LMICs

Cancer Care, E-health Solutions, and Challenges in LMICs

**Section 1: Barriers in Cancer Care within LMIC Context**

- What specific challenges or obstacles do you face when delivering optimal cancer care in Low- and Middle-Income Countries (LMICs), such as Jordan?

- 1.1. Could you elaborate with a case study where these challenges were particularly noteworthy?

**Section 2: Patient-Centric Hurdles**

- Given your clinical practice, what are the principal challenges cancer patients in LMICs face from diagnosis through to aftercare?

- 2.1. How do these challenges create repercussions for both patients and healthcare providers?

**Section 3: Digital Communication and Information Dissemination**

- What issues do you encounter in utilising e-health platforms for effective communication with cancer patients and survivors from diagnosis to aftercare?

- 3.1. What key digital content or features have you found to be particularly effective in enhancing patient outcomes?

**Section 4: Digital Elements of Supportive Cancer Care**

- What digital supportive care elements do you offer your patients from diagnosis to aftercare?

- 4.1. Can you offer an example where digital supportive care has positively impacted your patients?

**Section 5: Significance of Quality in Digital Communication and Information**

- From your perspective, what holds the most importance for cancer patients concerning the quality of digital communication and information they receive?

-5.1. Can you cite a situation that underlines this significance?

**Section 6: Key Patient Concerns in Specific Cancer Types**

- What stand out as the foremost concerns for patients with specific cancer types, such as breast or colon cancer?

- 6.1. How do these concerns shape your e-health solution strategies

**Section 7: Digital Patient Empowerment**

- What e-health solutions or digital tools have been effective in empowering patients in LMICs to be more engaged in their treatment and self-management?

- 7.1. Could you discuss a real-world application of such digital empowerment?

**Section 8: E-health as a Source of Support and Information**

- What do you consider to be the principal source of support for People Living With and Beyond Cancer (PLWBC) in LMICs?

- 8.1. How can e-health solutions improve the delivery of information to PLWBC?

- 8.2. Do you have any other observations, either challenges or best practices, in cancer care provision that you'd like to share?

- 8.3. Are there other concerns or best practices in information dissemination through e-health solutions you'd like to discuss?
